# Social brain circuitry and social cognition in infants born preterm

**DOI:** 10.1186/s11689-017-9206-9

**Published:** 2017-07-19

**Authors:** Angela Fenoglio, Michael K. Georgieff, Jed T. Elison

**Affiliations:** 1 Institute of Child Development, University of Minnesota, 51 East River Parkway, Minneapolis, MN 55455 USA; 2 Department of Pediatrics, Division of Pediatric Neonatology, University of Minnesota, 6th Floor East Building, MB630, 2450 Riverside Ave, Minneapolis, MN 55454 USA

**Keywords:** Prematurity, Social cognition, Social brain, Neurodevelopment, Neuroplasticity

## Abstract

Preterm birth is associated with an increased risk of adverse neurologic, psychiatric, and cognitive outcomes. The brain circuits involved in processing social information are critical to all of these domains, but little work has been done to examine whether and how these circuits may be especially sensitive to prematurity. This paper contains a brief summary of some of the cognitive, psychiatric, and social outcomes associated with prematurity, followed by a description of findings from the modest body of research into social-cognitive development in infants and children born preterm. Next, findings from studies of structural and functional brain development in infants born preterm are reviewed, with an eye toward the distinctive role of the brain circuits implicated in social functioning. The goal of this review is to investigate the extent to which the putative “social brain” may have particular developmental susceptibilities to the insults associated with preterm birth, and the role of early social-cognitive development in later neurodevelopmental outcomes. Much work has been done to characterize neurobehavioral outcomes in the preterm population, but future research must incorporate both brain and behavioral measures to identify early biomarkers linked to later emerging social-cognitive clinical impairment in order to guide effective, targeted intervention.

## Background

According to recent estimates from the Centers for Disease Control, approximately 1 in 10 infants born in the United States is delivered prior to 37 weeks of gestational age (GA) [1]. Worldwide, complications related to preterm (PT) birth are the leading cause of death in the neonatal period [2]. In the USA, prematurity accounts for 35% of all infant deaths, and is a principal cause of neurological disabilities in children [3]. Medical advances in recent decades have contributed to an increased survival rate in preterm infants [4], but this decreased mortality has been accompanied by an increase in morbidity [5]. In addition to medical complications [6, 7], infants born preterm are at an increased risk for a variety of adverse neurologic, psychiatric, and cognitive outcomes [8, 9].

Recent decades have seen an increasing emphasis on the pivotal role of early brain development for subsequent psychological functioning, and the recognized sensitive period has gradually shifted to encompass not only the infant and toddler years but also the time from conception to birth. Developmental science has long focused on the themes of “experience-expectant” and “experience-dependent” plasticity [10]. In this framework, experience-expectant plasticity refers to the idea that the human brain has evolved to expect certain inputs in order to select appropriate subsets of synaptic connections. In turn, a lack of these expected inputs (or inappropriate timing for these inputs) may lead to abnormal brain development. Experience-dependent plasticity, on the other hand, refers to the brain’s incorporation of environmental experiences unique to the individual. These inputs can occur throughout the lifespan and the nervous system is presumed to organize or specialize in response. Ostensibly, preterm birth can lead to alterations in both experience-expectant and experience-dependent development. Borrowing terminology from Luciana [11], PT-born infants are exposed to both “events of commission,” in the form of neurological or medical insult, and “events of omission,” in the form of less time spent in utero (accompanied by early exposure to the extra-uterine environment).

Given these atypical inputs, it is somewhat surprising that the majority of infants born PT develop various psychological capacities within the normal range, at least as measured by the off-the-shelf standardized measures that are frequently employed. Determining an appropriate “normal range” for comparison is complicated, however. Most often, PT-born infants and toddlers are compared to full-term (FT)-born children of the same “corrected” or “adjusted” age. These terms refer to postnatal age, corrected for degree of prematurity (e.g., an infant who was born at 32 weeks and is now 14 months old would be compared to an infant who was born at 40 weeks and is now 12 months old). Skills in some domains, such as sequences of gross motor development [12–14] appear to map to postmenstrual age (PMA; that is, gestational age plus postnatal age), which suggests a large degree of intrinsic developmental programming. However, in other domains, such as language comprehension [15], binocular vision [16], and some aspects of recognition memory [17], preterm (albeit healthy) infants seem to show some advancements relative to those born full-term. These differences in performance between preterm and full-term infants of the same adjusted age point to the importance of experience dependent learning in these domains, and it is readily conceivable that experience may be paramount when it comes to capacities, such as language, that are highly dependent on social interactions.

Critically, impaired social functioning is observed across a wide range of psychiatric and neurological disorders, including those that appear to be disproportionately prevalent in the preterm population. As conceptualized by Kennedy and Adolphs [18], the observable social interactions between individuals are termed “social behavior,” while “social functioning” refers to entrenched or consolidated patterns of interacting with others. In this schema, “social cognition” refers to the psychological processes underpinning social behavior, and the “social brain” comprises the brain regions that underlie social cognition. This putative social brain (see Table 1) encompasses a collection of highly distributed structures, circuits, and networks (including the amygdala, medial prefrontal cortex, the corpus callosum, the anterior and posterior cingulate cortex, and various regions within the temporal lobe) that have been identified as necessary for processing social information through various methodologies including lesion studies, neuroimaging, and behavioral assays. These areas have been consistently implicated in a number of neurological and psychiatric conditions, from schizophrenia [19, 20] and depression [21] to autism [22] and other neurodevelopmental disorders [23], and may be especially vulnerable to prematurity due to their topographical architecture and the temporal dynamics of emerging brain connectivity (as will be described later in the paper). Indeed, prematurity appears to predict a range of impairments in the social domain, from atypical gaze aversion [24] to difficulty establishing relationships [25] and overall lower social competence (see [26] for a meta-analysis).

**Table 1:** Selected social brain structures and networks

Region/network	Associated structures	Review	Associated social function/behavior
Amygdala		Adolphs [120]	Recognizing emotional expressions, social behavior towards conspecifics, reward learning
Default mode network	Posterior cingulate, medial prefrontal cortex, medial temporal lobe	Mars et al. [154]	Self-referential processing, self-projection, mentalizing/theory of mind
Corpus callosum		Paul et al. [130]	Social competence, introspection, judgment, planning, emotional communication
Uncinate fasciculus	Anterior temporal lobe, lateral orbitofrontal cortex, anterior frontal lobe	Olson et al. [140]; Von Der Heide et al. [138]	Social-emotional processing, social valuation

Despite growing evidence of prematurity’s psychosocial impacts, research into early social interactive and social cognitive behavior has largely been neglected. This knowledge gap is particularly surprising given the importance of social behavior in adaptive functional outcomes [27]. A brief summary of the history of neonatal research may help to contextualize the dearth of research on social-cognitive development. Initially and unsurprisingly, the primary concern for preterm infants was mortality. In the mid-1960s, an infant with a birth weight less than 1000 g had a survival rate of approximately 5% [28]. Medical advances such as mechanical ventilation, incubators with thermal regulation, intravenous feeding, and the administration of prenatal corticosteroids and pulmonary surfactant contributed to an enormous drop in mortality rates: by 1996, infants born at 750–1000 g had a survival rate of approximately 85% [29]. As is often the case, with this drop in mortality came a jump in morbidity; infants who previously would not have survived the first weeks of life were now at an increased risk of neurodevelopmental (as well as medical) disability [30]. As a result, interests shifted to predicting neurodevelopmental outcome. In the 1990s, as the level of care improved, rates of cerebral palsy and neurodevelopmental impairment increased [31]. Motor impairment was a consequence of great interest, for several reasons: it was visible, relatively common, well localized with regards to brain structure, and mapped well onto the adult stroke literature. Focus then expanded to cognitive morbidities [8, 32, 33], with a strong emphasis on general tests such as the Bayley Scales of Infant Development and the Griffiths Scales of Mental Development. At the same time, work in psychology and neuroscience was improving the anatomical localization of cognitive systems, and more tests of early cognitive ability became available. In the past few years, interest has shifted to the prevalence of attentional issues (and in particular, attention-deficit/hyperactivity disorder) in school-age children born PT [34–36]. This shift occurred partially as a consequence of longitudinal studies of PT-born children and adults; cohorts born very preterm (VPT; birth GA between 28 and 32 weeks) in the 1990s are now reaching adolescence and adulthood, and several large-scale studies [34, 37, 38] have been able to follow neonatal intensive care unit (NICU) survivors since birth. These studies have revealed more subtle impairments that may not be evident in infancy or early childhood, but present gradually and have detrimental effects in academic and professional domains. Atypical early social cognitive function may be one factor contributing to such subtle downstream effects.

The objective of this paper is to examine the extent to which the social brain may have particular developmental susceptibilities to insults (including events of both omission and commission) associated with preterm birth. Further, this paper describes the role that altered early social-cognition may play in later neurodevelopmental outcomes via longitudinal, cascading effects between developmental domains. Rather than a comprehensive review, the goal of this paper is a novel synthesis of research on brain and behavioral outcomes associated with prematurity, informed by knowledge of early social cognition and its role in development. A comprehensive understanding of social development requires crossing multiple levels of analysis, including the biological and behavioral. As such, this review will first address findings from the cognitive, psychiatric, and social-emotional domains that relate to specific behaviors and brain circuits tied to early social cognition. This is followed by a summary of the modest body of research into social-cognitive development in PT-born infants and children. Evidence suggests that multiple brain systems associated with social cognition are affected by preterm birth, and the next portion of the paper selectively summarizes findings from studies of structural and functional brain development in infants born preterm, with a focus on the distinctive role of the brain circuits involved in processing social information.

These findings, typically conceptualized as disparate in nature, collectively point to the possibility that alterations in the development of social brain circuitry may help to account for the atypical outcomes observed in the preterm population. This, in turn, implicates the putative prognostic value of assessing social behaviors and social brain circuits during infancy. Preterm birth serves as one common instantiating event linked to a variety of adverse outcomes, and strategic mental health intervention necessitates the comprehensive characterization of both adaptive and maladaptive neurodevelopmental trajectories. This work is crucial with regard to clinical questions—the “typical” trajectory of preterm social cognitive development must be characterized before it will be possible to identify atypical trajectories that might predict subsequent impairment. In addition, these studies will contribute to knowledge of more basic principles of neural plasticity, as they provide a unique opportunity to investigate the relative contributions of developmental programming and exposure to the social world.

### IQ and language

A number of studies have examined cognitive outcomes in children born preterm, at many time points and have used many different assessments. Typically, these follow-up measures are collected several years after birth, but recent work has indicated atypical cognitive development in the PT population even in the first years of life. One well-replicated finding is that PT-born individual score lower on measures of IQ throughout development than those born full-term [33, 39]. One domain commonly incorporated into such general developmental assessments is language. While language skills are typically conceived of as purely cognitive operations, there is a large corpus of evidence linking language development to early social communicative capacities. Infant joint attention and imitative behaviors, for example, have been found to predict various facets of both receptive and expressive language development [40–42].

Anderson [8] looked at medical, psychological, and developmental assessments conducted at 2, 5, and 8 years in a large cohort of very low birth weight (VLBW, birth weight < 1000 g) and/or VPT (GA < 28 weeks) infants, as well as a comparison cohort of term infants. The VLBW/VPT sample scored significantly lower than the control sample on full-scale IQ (as measured by the Wechsler Intelligence Scale for Children-Third Edition (WISC-III)), as well as all four subscales, including verbal comprehension. Reidy et al. [43] measured language abilities at age 7 in a cohort of 190 VPT (birth GA < 30 weeks) and 70 FT children using a battery of language-specific measures. The VPT/VLBW group had significantly lower scores on all measured language subdomains. A 2011 meta-analysis [44] incorporated 17 studies reporting both simple and complex language function outcomes (using the Peabody Picture Vocabulary Test and Clinical Evaluation of Language Fundamentals) in PT-born children between the ages of 3 and 12 years. This comprised a sample of 1529 PT-born (birth GA < 37 weeks) and 945 FT-born children. Here, the PT cohort scored significantly lower than the FT comparison group on both simple and complex language function, even after controlling for SES and major disabilities.

Taken together, these findings suggest that children born PT are more likely than those born FT to exhibit delays or impairments in cognitive development, and this risk appears to be correlated with degree of prematurity. What remains to be identified, however, are the mechanisms underlying these deficits. Imaging studies indicate that atypicalities in the development of specific brain structures may contribute to impairments in specific domains, but much work remains to be done. While none of these studies have looked directly at the relationship between social development and cognitive outcomes in the PT population, prior work has shown that early social behaviors (such as joint attention) contribute to complex competencies including language, and these competencies influence higher order cognition [45–48]. Thus, investigation of potential early social deficits may contribute to knowledge about the cognitive outcomes observed in PT-born infants.

### Social impairment in psychiatric outcomes

Preterm infants are also at an increased risk for maladaptive or clinically concerning behavioral problems consistent with various psychiatric classifications. As previously mentioned, dysfunction within the putative social brain has been associated with a vast range of psychiatric disorders, from schizophrenia [19, 20] to depression [21]. According to one recent estimate [49], children born very PT had three times the odds of meeting criteria for any psychiatric diagnosis by age seven when compared to full-term children. In this study, the most common diagnoses were anxiety disorders, attention deficit hyperactivity disorder (ADHD), and autism spectrum disorder (ASD). Similarly, Johnson et al. [50] followed up a sample of 307 children born prior to 26 weeks of gestation. At 11 years of age, parent and teacher reports suggested that VPT children had a threefold greater risk of showing symptoms consistent with a psychiatric disorder when compared to term classmates, with the most common disorders being ADHD, emotional disorders, anxiety disorders, and ASD.

While social impairment is widely recognized as a central component of ASD (as discussed in the following section), even ADHD is known to have associated social deficits. Some DSM criteria for ADHD directly implicate social functioning, such as “interrupting or intruding on others,” [51] and researchers have long observed impaired peer relationships [52, 53], friendships [54], and social communication skills [55] in children and adolescents with ADHD. It is sometimes hypothesized that these social difficulties may be more closely tied to comorbid disorders (such as oppositional defiant or conduct disorder) than to ADHD itself [56]. Although studies specific to social cognition are sparse, some evidence suggests that ADHD is associated with impairments in both complex social cognitive processes, such as theory of mind [57], and more basic processes, such as emotional face perception [58–60]. If there are social brain circuits that are particularly vulnerable to prematurity, identification of the developmental trajectories associated with these circuits may help to predict or prevent psychiatric symptoms.

### Autism spectrum disorders

Impaired social functioning (including deficits in social-emotional reciprocity, non-verbal communicative behaviors, and interpersonal relationships) represents a core feature of autism, and several studies have investigated a reputed link between preterm birth and ASD. Johnson et al. [61] assessed 219 survivors of birth prior to 26 weeks GA, as well as 153 FT classmates. At 11 years of age, parents completed a measure of ASD symptoms (the Social Communication Questionnaire (SCQ)) followed by a semi-structured diagnostic interview to diagnose ASD (the Development and Well-Being Assessment (DAWBA)). VPT children had a significantly higher frequency of ASD symptoms (as measured by the SCQ). After controlling for IQ, the PT cohort still showed significantly more impairment than the control group for social interaction, communication, and total scores, but not repetitive/stereotyped behavior. Sixteen (or approximately 7%) of the PT children and none of the FT children were diagnosed with ASD by 11 years. For comparison, a 2012 estimate places the prevalence of ASD in the general USA population at about 1.5% (Centers for Disease Control and Prevention (CDC), 2016), while the prevalence in siblings of children with ASD is estimated at approximately 11% [62].

The majority of the work pointing to an increased risk of ASD in the PT population has been limited to the use of screening measures (such as the SCQ and Modified Checklist for Autism in Toddlers (M-CHAT)), which are intended to identify all high-risk individuals; as such, these measures may overestimate the prevalence of ASD. For example, Limperopoulos et al. [63] investigated the prevalence of ASD in PT/VLBW infants at around 2 years of age, using a parent report checklist designed to screen for ASD symptoms (the M-CHAT), a standardized measure of functional status (the Vineland Adaptive Behavior Scale (VABS)), and the Child Behavior Checklist (CBCL). In this study, 26 percent of PT infants were flagged by the M-CHAT, and abnormal scores on that measure correlated with internalizing behavior problems (via the CBCL) as well as social and communication deficits (based on the VABS). Lower birth weight and gestational age were significantly correlated with abnormal MCHAT scores, though it should be noted that no FT infants were assessed. A few recent studies have used instruments designed specifically for diagnostic classification, rather than those designed to screen for risk. The Autism Diagnostic Observation Schedule (ADOS) is a direct, standardized, semi-structured observational assessment of the diagnostic features that define autism [64, 65]. Pritchard et al. [66] administered the M-CHAT and ADOS to groups of 2- and 4-year-old children born prior to 29 weeks GA. Of 169 individuals, 22 (13%) screened positive based on the M-CHAT, while only 3 (1.8%) met ASD criteria based on the ADOS.

There are several potential explanations for this notable discrepancy. As noted by Yaari et al. [67], parent-report questionnaires may index “developmental difficulties of the preterm phenotype” —in other words, the behaviors targeted by these questionnaires may indeed be symptoms of ASD, but some of them may also be related to medical conditions associated with prematurity, such as infection or neonatal illness [68]. Indeed, in a 2016 study [69] that used the M-CHAT at 2 years of age and the ADOS and ADI at 10 years of age in 827 extremely preterm (EPT) infants (birth GA < 28 weeks), the positive predictive value of the M-CHAT was approximately 20%. In other words, only one in five of the children who screened positive on the M-CHAT at age two had a diagnosis of ASD at age ten. In addition, impairments in vision and hearing were associated with higher misclassification rates. As the authors note, the sensory, motor, and cognitive impairments that are prevalent in the preterm population can all affect the validity of the M-CHAT. Additionally, use of the ADOS alone provides poorer *specificity* for ASD than its use in conjunction with the Autism Diagnostic Interview-Revised (ADI-R) [70]. The use of the ADOS and ADI-R in combination provides improved diagnostic validity than use of the ADOS alone [71].

All of this is to say that it remains unclear whether there is truly an increased prevalence of autism in the PT-born population. Nonetheless, these data support a rapidly expanding body of evidence suggestive of the etiologic heterogeneity of autism and raise interesting questions such as whether the proximal pathological mechanisms that result in the autistic-like symptoms in some preterm children are similar to or different from the proximal neural mechanisms that cause idiopathic autism. Again, clarity on this front requires further investigation of the effects of prematurity on the social brain.

### Social-emotional development

While autism presents a case with a clear emphasis on atypical social functioning, a number of recent studies indicate an association between preterm birth and atypical social-emotional development. In a study of 188 VPT (birth GA < 30 weeks or birth weight < 1250 g) and 70 FT (birth GA ≥ 37 weeks), parents of PT 2-year-olds reported significantly higher levels of internalizing behaviors and dysregulation, and lower social competence scores (as measured with the Infant-Toddler Social and Emotional Assessment (ITSEA)) [72]. Similarly, Cosentino-Rocha, Klein, and Linhares [73] asked mothers of 18–36-month-old children born PT (*n* = 44, birth GA < 37 weeks) and FT (*n* = 36, birth GA ≥ 38 weeks) to assess their children’s temperament (using the Early Childhood Behavior Questionnaire (ECBQ)) and behavioral problems (using the CBCL). PT children had significantly higher scores than the FT group on measures of high-intensity pleasure, perceptual sensitivity, and attention problems, and lower scores on discomfort, cuddliness, and attentional focusing. These social-emotional differences extend to deficits in the quality of parent-child interactions between preterm infants and their mothers, from comparatively lessened infant activity and responsivity [74] to reactions between infant irritability and maternal responsiveness [75] and lower levels of maternal involvement [76].

Rogers et al. [77] attempted to relate this increased risk of social-emotional issues to specific brain circuits, and found that social-emotional difficulties (as measured by the Strengths and Difficulties Questionnaire) at age five correlated with higher apparent diffusion coefficient (ADC; see below for explanation of diffusion MRI) in the right orbitofrontal cortex. This is a brain region that has been associated with social regulation and social cognition [78], and ADC typically decreases during development [79, 80], suggesting that a higher ADC may be representative of less mature white matter fiber bundles. Hille and Dorrepaal [25] assessed young adults born PT or VLBW as compared to peers from the general population. At around 19 years of age, the PT sample participated in less risk-taking behavior than their peers and did not report a higher rate of psychopathology, but males from the PT group did appear to have more difficulty establishing relationships. Critically, Ritchie et al. [26] conducted a systematic meta-analysis and concluded that children born VPT exhibit more peer problems, greater social withdrawal, and poorer social skills. The findings regarding prosocial behavior were mixed but the authors point out numerous limitations in the extant literature on social competence in children born PT, including the lack of longitudinal data, child or peer report, and conceptual models.

In summary, there is increasing evidence of prematurity’s specific effects on social behavior. As with the other domains discussed in this paper, the complex behavioral patterns involved in social competence as adolescents or adults have developmental roots in (and share neural circuitry with) early emerging social cognition.

### Social-cognitive development

Despite evidence of the close ties between cognitive, behavioral, social development, remarkably little research has been conducted on the topic of social-cognitive development in children born preterm. The capacity to deftly navigate the complex dynamics of social interaction, as observed in typical adults, has developmental roots in early social communication behaviors. This includes joint attention and reciprocal social behaviors that depend on rapid and efficient processing of social contingencies. Recent research has begun to map individual differences in these behaviors to individual differences in specific social brain circuits during the infant period [81].

Several groups have found evidence of early impairments in social development in infants born PT. In one study, PT infants averted gaze more frequently during social interactions at both 4 and 6 months [24]. In another study, PT infants performed more poorly than FT infants on measures of gaze following, joint attention, and behavioral requests at 14 months [82]. Telford et al. [83] tracked the gaze of PT-born (birth GA < 33 weeks) and FT 7-month-olds while viewing photographs of natural faces, natural face and scrambled face images vs. non-social images (phones, cars, and birds), and real-world scenes containing social or non-social content. The dependent measures were time to first fixation and looking time in areas of interest. The PT-born group showed shorter looking time durations to social content in all three tasks, suggesting less preferential attention for these stimuli.

One early behavior considered a cornerstone for social communication and later social-emotional development is joint attention (JA), which refers to the capacity to coordinate attention on an object with another person. In full-term infants, the abilities to respond to joint attention (RJA) and initiate joint attention (IJA) show specific patterns of age-related growth between 9 and 18 months of age. Additionally, RJA at 12 months and IJA at 18 months predicted language abilities at 24 months, after controlling for cognitive development [84]. These skills also underpin other higher order competencies such as theory of mind [48].

One early study investigating joint attention behaviors in PT and full-term 6-month-olds [85] found no significant differences between groups. However, the experimental context, a 2-min videotaped naturalistic interaction between mother and infant, was fairly uncontrolled. Further, the timing of this assessment may not have been ideal for detecting differences given that relevant joint attention behaviors tend to emerge later in life, between 6 and 9 months. Another more recent study [86] compared gaze following (a critical precursor to joint attention) in groups of PT-born 7-month-olds, PT-born 10-month olds, FT-born 4-month-olds, and FT-born 7-month-olds. Thus, individuals in the PT cohort could be matched to FT infants on either postmenstrual age (time in utero + time ex utero) or on chronological age (time ex utero). The impetus for this comparison was the idea that a longer duration of exposure to the rich ex utero social environment could potentially accelerate social development in infants born PT. Gaze following was measured in two contexts: in one, adults cued the infant to orient their gaze to one toy or another using both head and eye movements, and one in which the adult cued using eye movements alone. The 7-month-old PT infants performed like 7-month-old FT infants (with whom they shared chronological but not postmenstrual age) in both tasks, suggesting that time functioning in the social world, and not just time since conception, might affect the development of social behavior.

### Development of social brain circuitry

An accumulating body of work has investigated atypical structural and functional brain development in infants born preterm. In a recent study of 325 infants born prior to 32 weeks of gestation or with a birth weight of less than 1250 grams, approximately one third showed some form of brain injury, with approximately 10% showing evidence of severe brain injury [87]. Meanwhile, estimates of the rate of neurobehavioral impairment in a similarly-sized cohort of PT and extremely low birth weight infants are closer to 50% [88]. Thus, the occurrence of brain injury does not fully account for the incidence of adverse neurodevelopmental outcomes seen in the PT population. Traditionally, studies have focused on the increased prevalence of injury in the PT-born population. More recently, however, there is growing recognition that the altered developmental trajectories observed in this group are not fully accounted for by events of commission (such as white matter injury) but may also hinge on “events of omission” in the form of a lack of typical environmental inputs, suggesting the body’s neurodevelopmental processes may be programmed to expect 40 weeks in the womb. In other words, these atypicalities are likely influenced by a complex interplay of biology (in the form of developmental programming) and experience (in the form of early exposure to the ex-utero environment).

#### Altered gray and white matter volumes in social brain regions

Magnetic resonance imaging (MRI) studies show that the most common structural observations in PT infants include diffuse white matter (WM) and gray matter (GM) abnormalities. Periventricular leukomalacia (PVL) is characterized by both focal and diffuse lesions to the brain’s white matter. Inder et al. [89] collected quantitative volumetric MRI in preterm infants (birth GA < 32 weeks) with prior evidence of PVL. At term, these infants displayed a reduction in cerebral cortical (but not subcortical) gray matter, along with a reduction in the volume of total brain myelinated (but not unmyelinated) white matter. PVL was, until recently, the most commonly diagnosed brain injury in PT infants. The recent decrease in the prevalence of cystic PVL is thought to be associated with several factors, including reductions in the duration of mechanical ventilation and the incidence of bacterial sepsis [90]. It has since been recognized, however, that white matter damage often occurs in the absence of the cystic regions of necrosis seen in classic PVL [91, 92]. A later study [93] reported reduced cerebral cortical gray matter volume and deep nuclear gray matter volume in 119 preterm infants (birth GA ≤ 32 weeks, birth weight ≤ 1500 g), as compared to 21 full-term infants, and found that these abnormalities correlated with moderate to severe neurodevelopmental disability at 1 year of age. Brown et al. [94] imaged a cohort of very preterm (VPT, birth GA < 30 weeks or birth weight < 1250 g) infants and found that lower composite neurobehavioral scores at term were related to the degree of white matter abnormality observed via MRI, and correlated most strongly to abnormal WM signal and reduced WM volumes, rather than the grade of GM abnormality. In aggregate, these findings suggest atypical gray and white matter development and associated behavioral abnormalities occur even in PT infants who do not meet criteria for canonical insults such as PVL. Neurodevelopmental research has traditionally been focused on infants with identifiable injury or severe symptomatology, but there is increasing recognition of the need to identify infants who may manifest more subtle impairments.

The brain undergoes rapid and dynamic development during the latter two trimesters. In terms of gross structure, total brain volume increases and ventricle size decreases during the second trimester, and while major fissures begin to emerge, the cerebral surface remains largely smooth until the end of the second trimester [95]. A small amount of cortical folding occurs by 25 weeks, and folding intensifies in the third trimester [96]. This folding occurs in an orderly fashion, with many primary sulci forming during the second trimester, and tertiary sulci appearing during the third trimester and after term [97]. Sulci and fissures emerge in a posterior to anterior fashion: the parieto-occipital, calcerine, and cingulate sulci around 20–22 weeks GA; the central, interparietal, and superior temporal sulci by 25 weeks GA, and the precentral, post-central, superior frontal, and middle temporal sulci around 24–28 weeks [98]. Between 18 and 40 weeks GA, fetal white matter volume shows a 22-fold increase, along with a 21-fold increase in cortical gray matter and a 10-fold increase in deep subcortical structures [99]. Presumably, birth prior to 40 weeks may disturb the typical progression of neural circuit organization. Structural MRI studies have indeed shown abnormalities in specific structures and regions in the preterm brain, including areas associated with the putative social brain. The majority of these studies have imaged former PT infants at school age or later. This later-stage imaging (see Box 1) introduces difficulties in interpretation—while this body of work suggests lifelong consequences for a portion of the PT-born population, the substantial interim between birth and assessment introduces difficulty in specifying the contributions of prematurity, per se, as opposed to potential downstream or tangential effects. For this reason, this portion of the review will focus on data collected during infancy, rather than imaging acquired later in childhood or in adulthood.

As mentioned above, the set of structures associated with social cognition is widely distributed, and emerging evidence suggests that prematurity is associated with altered brain volumes in many of these regions. Thompson et al. [100] compared 202 preterm (birth GA < 30 weeks and/or birth weight < 1250 g) and 36 term infants (scanned at term equivalent age (TEA)) and found that PT infants showed reduced cortical gray matter and unmyelinated WM volumes in orbitofrontal regions. Similarly, Ball et al. [101] collected scans from 71 preterm (birth GA < 136 weeks) infants at TEA and found that degree of prematurity was associated with volume reduction in the orbitofrontal lobe. The orbitofrontal cortex seems to be a key for self-monitoring [102] and emotional self-regulation [103], amongst many other behaviors.

In the parietal region, Ball et al. [101] also found that increasing prematurity was associated with reduced posterior cingulate cortex (PCC) volume. This finding was echoed by Gousias et al. [104], who compared scans from 15 PT infants (<36 weeks GA, imaged at TEA) and 5 FT infants and also found that PT infants had relatively smaller PCC volumes. The PCC is thought to play a crucial role in the so-called “default mode network” (DMN) [105]. This network includes the PCC, medial prefrontal cortex (mPFC), and medial temporal lobe (MTL) and several studies have pointed to its role in social-cognitive processing [106, 107].

Significant volumetric differences have also been observed in the occipital region in the brains of PT infants. Peterson et al. [108] scanned 10 PT (birth GA not specified) and 14 term infants once between 34 and 42 weeks GA and calculated volumes for cortical gray and white matter parcellated into 8 subregions. In this study, PT infants were observed to have reduced GM volumes in the parieto-occipital and inferior occipital cortices. Similarly, Thompson et al. [100] found that PT infants showed reduced deep nuclear gray matter in the parieto-occipital region. The extrastriate body area and occipital face area, which would likely be captured in the “inferior occipital” subdivision, have been implicated in perceiving human bodies and body parts [109] and accurate face perception [110], respectively. The area designated as “parieto-occipital” in these studies also may have been large enough to capture the temporoparietal junction, an area associated with numerous aspects of social cognition including theory of mind [111], empathy [112], and moral judgments [113].

Many of the structures associated with the social brain are concentrated in the temporal lobe, including the amygdala [114], the superior temporal sulcus [115] and superior temporal gyrus [116], the fusiform face area [117], and the temporal pole [116], among others. In Gousias’ study [104], several significant differences were pinpointed in the temporal region. Here, PT infants had relatively smaller volumes in the middle and inferior temporal gyri, as well as the anterior temporal lobe. Interestingly, in this sample, the PT group (as compared to the FT group) showed larger volumes in the amygdala, as well as the left superior temporal gyrus (though it should be noted that none of these group differences remained significant after correcting for multiple hypothesis testing). The amygdala is one region consistently implicated in processing social information, including emotional responses [118], detection of socially salient information [114], and social affiliative behavior [119] (see [120] for a review of amygdala function). The temporal lobe has also been a region of strong interest in follow-up imaging conducted after infancy (see Box 1).

While volumetric findings can admittedly be difficult to interpret, the research reviewed above suggests that preterm birth is associated with atypical cortical and subcortical development. This altered development could be a result of disruptions to the typical developmental processes occurring during the second and third trimesters. These alterations in both gray and white matter appear in several brain regions believed to underlie processing of social information.

#### Abnormalities in social brain microstructure

Much of the most recent work on preterm brain development has used a method called diffusion weighted imaging (DWI) to investigate microstructural development.

Diffusion weighted imaging (DWI) measures the restricted diffusion of water molecules. The rate of diffusion is affected by tissue structures. For example, water moves more readily *along* axonal bundles than *perpendicular* to them and diffuses isotropically in cerebrospinal fluid. The directionality of anisotropic diffusion of any given voxel can be characterized by applying a tensor model (i.e., diffusion tensor imaging or DTI). By providing information about the spatial distribution of water diffusion in a given voxel, diffusion imaging can serve as a tool for quantitatively characterizing brain microstructure. Common diffusion metrics include axial diffusivity, radial diffusivity, and mean diffusivity. Axial diffusivity (AD) refers to diffusion parallel to the principle eigenvector of the three-dimensional voxel, while radial diffusivity (RD) denotes diffusion in the direction orthogonal to the principle eigenvector. Mean diffusivity (MD) or apparent diffusion coefficient (ADC) represents the average of the two fractional anisotropy (FA) reflects the degree of diffusion along the principle axis relative to the other directions. FA is a scalar metric with values ranging from 0 (unrestrained or “isotropic” diffusion) to 1 (completely constrained or “anisotropic” diffusion). Opinions differ somewhat as to which aspects of the brain’s microstructural architecture are reflected best by each measure. It is thought that MD is most closely related to overall water content in the brain [121], while FA represents an index of the brain’s microarchitecture that is influenced by axonal density, axonal diameter, cell membrane and microtubule composition, myelination, and dendritic cytoarchitecture [80, 121–123].

In terms of gray matter, postmortem studies of fetal brains show an increase in regional cortical FA from 15 to 28 weeks of gestation, followed by a decrease through 36 weeks [124]. This finding is consistent with the migration of neurons from the germinal matrix, as it is thought that the bulk of cortical neurons migrate along radially organized glial fibers early in gestation (Rakic, 1988, 2003; Rakic and Kornack, 2001; as cited in [124]), perhaps contributing to the increased cortical FA observed prior to 27 weeks. At this point, these glial cells retract (Volpe, 2001; Rakic 2003; as cited in [124]), ostensibly resulting in a decline in cortical FA. Thus, gray matter microstructure undergoes significant change during the period of gestation disrupted by preterm birth.

McKinstry et al. [122] attempted to characterize water diffusion in vivo in neonatal cortical gray matter by imaging 24 PT and FT infants (birth GA 26–41 weeks) in the first 36 h of life. These studies found that (similar to Huang et al. [95]) the cerebral cortex shows a pronounced radial organization by 26 weeks of gestation, but this radial organization disappears by term, as evidenced by a decrease in averaged water diffusion coefficient. Ball et al. [123] imaged preterm infants (birth GA 23–35 weeks), 47 of whom were scanned once, and 9 of whom were scanned twice. Between 27 and 46 weeks post-conception, a decrease in cortical FA was observed until week 38. Higher initial FA values and rates of change were seen in gyri, frontal and temporal poles, and parietal cortex, with lower values and rates of change in sulcal, perirolandic, and medial occipital cortex. Kersbergen et al. [125] looked at longitudinal change in FA in 122 distinct regions of the brain in infants between 30 and 40 weeks of postmenstrual age. The sample included infants born prior to 28 weeks of gestation and was limited to individuals without cerebral injury and with a normal neurodevelopmental outcome (as characterized at 15 months of age with the Griffiths Mental Development Scales), who were scanned longitudinally around 30 weeks of gestation and again at term equivalent age. In cortical brain areas, frontal areas showed a slight increase in FA, while temporal-occipital areas exhibited a slight decrease in FA. The majority of brain regions showed a clear decrease in mean, radial, and axial diffusivity, though the degree of decrease was heterogeneous across regions. Similar to the pattern of FA change, increases in MD, AD, and RD were seen in the occipital, temporal, and frontal regions. As described above in the context of structural imaging, the putative social brain encompasses cortical areas spread across the brain. As such, microstructural alterations in these areas (as indexed, in this case, by altered diffusion metrics) might contribute to atypical patterns of social cognitive development.

White matter has been of particular interest in the realm of preterm brain development, for multiple reasons. As described earlier, it appears to be acutely vulnerable to insult. DWI provides especially important information about white matter development during the perinatal period in the early stages of myelination [79]. Studies of postmortem fetal brains suggest that white matter fibers transition from a dominant radial organization around 15 weeks of gestational age to a more laminar and radial architecture through the duration of the second trimester [95], Meanwhile, white matter tracts considered crucial to early social-cognitive behaviors (including the corpus callosum, the uncinate, and the inferior longitudinal fasciculus) undergo rapid change from 13 to 22 weeks of gestation [95]. More generally, imaging from fetal brain specimens and full-term neonates [95, 126] indicates that limbic fibers (including the stria terminalis, fornix, and cingulum) are well formed by 19 weeks of gestational age. Association fibers (including the uncinate fasciculus, inferior fronto-occipital fasciculus, and superior and inferior longitudinal fasciculi) seem to develop relatively more slowly [95]. Based on three-dimensionally reconstructed WM tracts of fetal specimens, the uncinate is visible by 15 weeks of gestation, but the superior longitudinal fasciculus is still not prominent by term [95]. White matter FA appears to increase rapidly from term through the first 2 years of life, at which point it slows but continues to increase through childhood [79, 127].

Critically, several of the tracts developing largely in the second and third trimesters have been consistently implicated in various aspects of social processing. The corpus callosum is visible in rudimentary form by the twelfth week of gestation [128], but undergoes exuberant proliferation during the third trimester followed by a period of axonal pruning until the second month post-term [129]. Much of the work investigating the role of the corpus callosum in social functioning comes from studies of individuals in whom the fibers of the corpus callosum fail to develop, a condition known as agenesis of the corpus callosum (AgCC). Parents of children with AgCC report impairments in social competence, introspection, social judgment and planning, and poor emotional communication [130]. Abnormalities of the corpus callosum have also been associated with the range of social deficits observed in autism, specifically measures of social responsiveness [131]. One study of corpus callosum diffusion at TEA in 58 PT infants (divided into groups of 23–25, 26–29, and 30–33 weeks birth GA, all without apparent WM lesions) found that lower birth GA was associated with lower FA in the posterior corpus callosum, such that FA decreased linearly with birth GA [132]. Another study using DTI in 44 PT (birth weight between 600 and 1250 g) and 41 FT adolescents found that prematurity was associated with lower FA in multiple regions including the splenium of the corpus callosum [133]. Eikenes et al. (2011) also found reduced FA in the corpus callosum of 49 VLBW (birth weight < 1500 g) PT-born 12-year olds, when compared to 59 term-born controls [134]. A recent meta-analysis [135] incorporated 232 original studies, for a total of 513 PT-born children, adolescents, and young adults and 309 healthy controls. The analysis used an activation likelihood estimate (ALE) procedure to identify regions of abnormal FA in the corpus callosum. The ALE analysis yielded 11 regions of decreased white matter FA in the PT versus FT group, including the bilateral splenium of the corpus callosum, the bilateral external capsule, the left superior fronto-occipital fasciculus, the left posterior thalamic radiation, the right superior longitudinal fasciculus, the genu of the corpus callosum, the left cingulum, the left posterior corona radiata, and the left posterior limb of the internal capsule (also seen in [136]). Four regions of increased FA were observed, including the bilateral posterior corona radiata and the anterior corona radiata. The authors interpret these findings to mean that the mechanisms underlying alterations in FA in the preterm brain may vary during different stages of white matter development. The areas of increased FA were smaller than the areas of decreased FA (192 and 4360 voxels, respectively), and the authors note that the findings of decreased FA are more consistent with prior work. The regions of increased FA seem harder to explain; it is suggested that these increases may in fact be due to differences in methodology between studies.

The uncinate is a bidirectional long-range pathway that extends from the anterior temporal lobes and amygdala to the lateral orbitofrontal cortex [137]. While the exact function of the uncinate is under debate [138], it serves to connect areas believed to be vital to various social-cognitive functions, and has been associated with behaviors such as joint attention [81] and sensitivity to social rewards [139]. Abnormalities in the uncinate have been associated with various psychiatric disorders (see [140] for a review), and it has been hypothesized that the slow time course of uncinate development may make it particularly susceptible to perturbation contributing to social-emotional problems. There has been little investigation of the uncinate in PT infants, but in addition to their findings related to the corpus callosum, Eikenes et al. [134] and Mullen et al. [133] found reduced FA in the uncinate of adolescents born preterm.

Recent years have seen a striking increase in work investigating diffusion in the PT brain, and as of yet there is little consensus as to the precise structures or circuits most often affected by prematurity. As Li [135] notes, this may be due at least in part to differences in methodology, including the population of interest, the time at which images are collected, and the manner in which brains are segmented into regions or networks. What is clear is that even uninjured PT-born infants show differences in brain connectivity, both structurally and functionally (as described below).

#### Altered functional connectivity

One burgeoning area of research involves functional imaging in preterm infants. The bulk of this work has been conducted with adolescents or adults born preterm, in which case it is challenging to draw specific conclusions given the amount of time lapsed, but some groups have attempted to characterize functional activity closer to the time of birth. As described in the diffusion imaging section, prematurity has been found to be associated with alterations in the brain’s connectomic architecture. Another way to characterize the development of the brain’s neural circuitry is to examine functional connectivity through methods such as resting state fMRI (functional magnetic resonance imaging). Echoing the aforementioned findings related to structural brain changes in the third trimester, this is also a time of great change in the organization and connections in the brain’s functional networks. In a cohort of 40 preterm and full-term infants (birth GA 25 to 41 weeks) imaged between 31 and 42 weeks PMA, higher PMA was associated with greater functional connectivity [141]. There were also regional differences, such that age-dependent increases in connectivity were greater in primary sensory and motor regions and lesser in default mode and executive-control regions. Doria et al. [142] collected resting state fMRI (rs-fMRI) data in 70 infants born between 29 and 43 weeks of gestation. Several networks (visual, auditory, somatosensory, default mode, frontoparietal, and executive control) were examined and appeared to emerge at differing rates, though all showed rapid change during the period from 30 to 40 weeks PMA. Similarly, van den Heuvel et al. [143] imaged 27 PT infants (birth GA 24–29 weeks) at 30 and/or 40 weeks PMA and found evidence of resting state networks by 30 weeks PMA. He and Parikh [144] also observed significant increases in functional connectivity strength in a large set of resting-state networks between 32 and 52 weeks PMA in a sample of 34 VPT infants (birth GA ≤ 32 weeks). Prematurity is associated with widespread alterations in functional connectivity (see [145–147] for comprehensive review). Findings reveal impaired cerebral lateralization [148, 149], atypical thalamocortical connectivity [150], and reduced connectivity between “rich” nodes; a set of cortical regions which are typically highly connected [151]. These findings are supported by machine learning studies which have used whole-brain functional connectivity to successfully classify infants as either preterm or full-term [152], and even to estimate birth GA [153].

One focus of functional research has been the aforementioned “default mode network,” (DMN) which has been consistently implicated in higher-order social cognitive behaviors such as self-referential processing and self-projection [154]. Fransson et al. [155] collected resting state data around term age from 12 preterm (between 24 and 27 weeks birth GA) and did not find evidence of an infant equivalent of the DMN. It is worth noting, however, that this study did not include a comparison group of FT-born infants. Smyser et al. [156] observed precursors to the DMN in 10 FT but not 53 VPT (birth GA 26–28 weeks) infants, scanned longitudinally from 26 to 40 weeks PMA. A later study [153] included resting state data from 50 PT-born (23–29 weeks birth GA, without moderate-severe brain injury) compared to 50 FT-born infants, all scanned at TEA. Using machine learning algorithms and 214 regions of interest, they were able to distinguish PT and FT-born infants with 84% accuracy. Here, the default mode network contributed to successful categorization, suggesting that preterm birth contributes to altered functional connectivity even in the absence of brain injury. Similar results have been found with PT-born individuals later in life: at 36 months of age, PT-born children show relatively weaker connectivity between resting state networks including the DMN [157]. One recent study also examined functional connectivity in the amygdala of 65 FT (birth GA > 36 weeks) and 57 PT (birth GA < 30 weeks) infants imaged at TEA [158]. Here, functional connectivity patterns in the PT and FT groups were similar to those of older children and adults, though prematurity was associated with reduced magnitude of activation.

Collectively, these imaging findings suggest an array of maturational changes throughout the brain during the second and third trimesters that might be interrupted or altered by preterm birth. These results indicate two potential sources for the unique vulnerability of the PT brain. First, prematurity is associated with an increased risk of specific brain insults such as intraventricular hemorrhage, white matter damage, and hypoxic-ischemic injury. Secondly, and perhaps more critically, the PT brain shows atypicalities in the extensive network connectivity seen by term, and atypical early life experience—even in the form of early exposure to the extra-uterine environment—may result in these connections forming abnormally. Prematurity appears to be associated with altered brain architecture and connectivity in a variety of cortical and subcortical brain regions, including areas and networks traditionally implicated in social-cognitive processing. However, there are few (if any) biomarkers relating specific brain abnormalities to the long-term cognitive, psychiatric, or social outcomes for which preterm infants are at an increased risk.

## Conclusions

The first years after conception are a critical period of development, showing rapid change in both brain and behavior. In vivo and ex vivo imaging studies suggest that the third trimester is a critical period for brain development, and preterm birth is associated with alterations in brain structure, function, and connectivity. PT-born infants show atypical cortical and subcortical development, presumably resulting from disruptions to typical maturational processes across the brain, including several of the regions implicated in social processing. These neural anomalies have been linked to abnormal cognitive development in the PT population in infancy as well as childhood and early adulthood. PT-born individuals also appear to be at an increased risk of adverse neurobehavioral outcomes, from a relatively higher incidence of anxiety disorders and attentional impairment to abnormal social-emotional development and a putative increased risk of autism. The social brain plays a crucial role in all of these domains of impairment, yet little work has been done to investigate whether and how these neural circuits may be especially sensitive to both the events of omission and events of commission associated with the premature brain. Thus, in order to do justice to the complexity of development, research must incorporate both brain measures and assessment of social-cognitive behavior.

This begs the question of how and why research into social-cognitive development in the PT population fell so far behind studies of development in other domains. One explanation might be that social processes are more difficult to localize in the brain, but as detailed in this paper our knowledge of the relevant neural circuitry has expanded greatly in the past decade. Another justification might be that social-cognitive development is harder to assess across time. Indeed, specific and informative measures of social behaviors have lagged behind those in the purely cognitive domain, but this social cognitive toolbox is growing. More broadly, there seems to be a fundamental failure to understand how social impairments or delays, particularly in the presence of normal IQ, can compromise quality of life for both children and adults. Our knowledge is limited to the extent that we cannot address something as basic as whether social-cognitive milestones are mediated more by postmenstrual or postnatal age. It is possible, in fact, that different areas of the same circuit may be driven by postnatal and postmenstrual age. In this case, prematurity could lead to a disconnect in maturation rates between these areas. Such brain dysmaturity [164] may be one cause of the adverse neurodevelopmental outcomes observed in the PT population.

Following the research reviewed here, there are a number of areas ripe for exploration. While more and more research groups are working to image the brain during infancy, much of the work linking specific biomarkers (such a structural or functional brain abnormalities) to behaviors (such as language delay, or externalizing behaviors) focus on outcomes measured after interludes of years or even decades. Developmental scientists have a number of tools that can be used to measure both brain and behavior in infancy, which could help to disentangle potential cascading effects. This is not to say that measurement at a single time point is ideal; rather, the ability to characterize developmental trajectories hinges on a need for longitudinal data. In addition, the identification of atypical trajectories requires knowledge of the *typical* trajectory of preterm development, which pinpoints another gap in the field: there is currently a paucity of data from the “normative” PT population. Little work has been done to characterize brain and behavioral development in lower-risk PT infants and children, such as those born moderate to late preterm. Rather, most of the studies of preterm development draw from samples of the very preterm and/or very low birth weight.

While much effort has been invested into characterizing adverse neurobehavioral outcomes in the PT population, there are few biomarkers relating specific brain abnormalities to the adverse outcomes for which preterm infants are at an increased risk. Many of these outcomes may have developmental antecedents in early emerging social cognition, which in turn may reflect abnormal social brain circuit function. More broadly, we do not fully understand how individual differences in social functioning map to individual differences in development of the neural circuitry of the social brain, and preterm infants may serve as a model population for characterizing these associations. The PT brain is not only quantitatively but also qualitatively different from the brain of a FT infant. Researchers now have the means, the numbers, and certainly the rationale to study the social brain in infants born PT. Future work should make use of behavioral measures of social functioning linked to specific neural circuitry in order to identify the specific brain circuits that are at risk following PT birth and may benefit from targeted behavioral interventions. Such work will allow for the characterization of differential trajectories of social cognitive and brain development in infants born preterm, and the identification of early social cognitive behaviors and brain signatures related to later emerging clinical impairment. Ultimately, targeted assessment of the social brain in infancy has the potential to improve the lives of a substantial proportion of infants born preterm.

Box 1 Kesler et al. [159] imaged 73 PT-born (birth weight 600–1250 g, GA estimated using the Ballard score [160]) and 33 FT-born 8-year olds and found that the PT group had relatively larger volumes in the parietal and frontal gray matter, occipital horn, and ventricular body, as well as relatively smaller temporal and subcortical gray matter volumes. Peterson et al. [161] imaged children who had been born preterm (birth weight 600–1250 g, GA estimated via Ballard score) when they were corrected to 8 years of age and were found that (compared to term controls), the PT cohort had significantly reduced cortical volumes in regions including the sensorimotor and premotor areas, the midtemporal area, the parieto-occipital cortex, and sebgenual cortex. Subcortically, significantly smaller volumes were seen in the amygdala, cerebellum, basal ganglia, hippocampus, and corpus callosum, while *larger* volumes were observed in the occipital and temporal horns of the ventricles.

Northam et al. [162] investigated interhemispheric temporal lobe connectivity (via MRI and DTI) and language abilities in 50 PT-born (birth GA < 33 weeks) and 30 FT-born adolescents. Results suggested that 38% of PT-born adolescents had some language impairment, and this impairment was associated with reduced volumes in the posterior corpus callosum. In the DTI study from Mullen et al [133], FA in the bilateral uncinate fasciculi correlated with scores on a semantic language task in the PT (but not FT) cohort.

Lawrence et al. [163] collected functional and structural MRI data in a cohort of young adults (around 20 years of age) who had been born preterm (birth GA < 33 weeks), as well as a set of full-term controls. This study used a cued motor task, in which participants were instructed to keep a joystick still when presented with the word “rest,” and to move the joystick in a randomly chosen direction when presented with the word “move.” While task performance (as measured by reaction time) was comparable between the VPT and control groups, the VPT group showed greater activation in a region containing the right cerebellum as well as the lingual, parahippocampal, and middle temporal gyri, along with decreased GM volume in the right superior frontal/premotor cortex and left middle temporal gyri. These studies provide evidence that atypicalities persist even decades after birth in adolescents and adults born preterm.

## Abbreviations

AD: Axial diffusivity
ADC: Apparent diffusion coefficient
ADHD: Attention deficit hyperactivity disorder
ADI-R: Autism Diagnostic Interview-Revised
ADOS: Autism Diagnostic Observation Schedule
AgCC: Agenesis of the corpus callosum
ALE: Activation likelihood estimate
ASD: Autism spectrum disorder
CBCL: Child Behavior Checklist
DAWBA: Development and Well-Being Assessment
DMN: Default mode network
DSM: Diagnostic and Statistical Manual of Mental Disorders
DTI: Diffusion tensor imaging
DWI: Diffusion weighted imaging
ECBQ: Early Childhood Behavior Questionnaire
EPT: Extremely preterm
FA: Fractional anisotropy
fMRI: Functional magnetic resonance imaging
FT: Full-term
GA: Gestational age
GM: Gray matter
IJA: Initiating joint attention
ITSEA: Infant-Toddler Social and Emotional Assessment
JA: Joint attention
M-CHAT: Modified Checklist for Autism in Toddlers
MD: Mean diffusivity
mPFC: Medial prefrontal cortex
MRI: Magnetic resonance imaging
MTL: Medial temporal lobe
NICU: Neonatal intensive care unit
PCC: Posterior cingulate cortex
PMA: Postmenstrual age
PT: Preterm
PVL: Periventricular leukomalacia
RD: Radial diffusivity
RJA: Responding to joint attention
SCQ: Social Communication Questionnaire
TEA: Term equivalent age
VABS: Vineland Adaptive Behavior Scale
VLBW: Very low birth weight
VPT: Very preterm
WISC-III: Wechsler Intelligence Scale for Children-Third Edition
WM: White matter
